# Methylene Blue Absorption in Sentinel Lymph Node Biopsy for Early Breast Cancer after Neoadjuvant Chemotherapy

**DOI:** 10.31557/APJCP.2020.21.6.1767

**Published:** 2020-06

**Authors:** Terry Soebhi, Kristanto Yuli Yarso, Farida Briani Sobri, Ida Bagus Budhi

**Affiliations:** 1 *Faculty of Medicine, Sebelas Maret University, Surakarta, Indonesia.*; 2 *Department of Surgery, Oncology Division, Faculty of Medicine, Sebelas Maret University, Surakarta, Indonesia. *; 3 *Department of Surgery, Oncology Division, Metropolitan Medical Center Hospital, Jakarta, Indonesia. *; 4 *Department of Surgery, Digestive Division, Faculty of Medicine, Sebelas Maret University, Surakarta, Indonesia. *

**Keywords:** SLNB, Neoadjuvant, chemotherapy

## Abstract

**Introduction:**

Chemotherapy is claimed to cause lymphatic drainage damage because of the tumor cell’s apoptosis process. This event might cause decreased marker (radioactive solution and/or blue dye) absorption on sentinel lymph nodes (SLN). In this study, the researchers used methylene blue only and wished to evaluate the methylene blue absorption of the SLNB procedure on early-stage breast-cancer patients after neoadjuvant chemotherapy (NAC).

**Materials and methods:**

The method used was the historical cohort study conducted from 2016-2019 in Indonesia. Samples were collected from 117 patients of stage I and II breast cancer with clinically negative axillary lymph nodes, who were then grouped into post-NAC and no-NAC (control group), in which SLNB procedures were conducted on the two groups by using single-method methylene blue. The results of methylene blue absorption were then analyzed by the Chi-square hypothesis test.

**Results:**

From the total of 564 early-stage patients who were referred to surgical oncologists, 117 patients were found to meet criteria of inclusion, consisting of the control group (52 patients) and the post-NAC group (65 patents). Of 65 patients who had undergone NAC treatment and SLNB procedure, it was found that 40 patients (61.5%) showed positive blue SLN. Of 52 pre-NAC breast-cancer patients, it was found that 47 patients (90.4%) showed methylene blue absorption on SLN with the p-value of 0.000 (P<0.05, significant). The relative risk value amounted to 0.522. Post-NAC patients had a tendency of decreased absorption of methylene blue.

**Conclusion:**

Neoadjuvant chemotherapy can cause the decrease of methylene blue absorption on SLNB procedure.

## Introduction

Axillary lymph nodes play a considerable role as the prognosis and the basis in determining therapies for treating early-stage breast cancer (Newman, 2007). Axillary lymph nodes mapping technique can be performed by using Axillary Lymph Node Dissection (ALND) or Sentinel Lymph Node Biopsy (SLNB). On the ACOSOG Z0011 trial, it is stated that the SLNB procedure is nearly the same as ALND in terms of recurrence and mortality on early-stage breast-cancer patients, subsequently treated with breast conservation surgery and continued with radiotherapy treatment (Giuliano et al., 2011). The previous SLNB study of Brahma et al., (2017)in Indonesia, the identification rate without neoadjuvant chemotherapy using methylene blue only was 91.7%. A study of SLNB after NAC on a single-institution study from 2000 to 2004 reported that the significant variability in the SLN identification rate was found 89%, with the average FNR of around 11% (Breslin et al., 2000). The application of SLNB in neoadjuvant chemotherapy patients with breast cancer may identify patients who do not require an axillary dissection.

Neoadjuvant chemotherapy (NAC) on axillary lymph nodes can cause gland atrophy. Microscopically, lymph nodes show lymphocyte disappearance, occurrence of fibrosis, and histiocytic collection (Fan, 2009). It is suspected that chemotherapy may cause lymphatic drainage damage by inducting fat degeneration because of the tumor cell’s apoptosis process Cohen et a., (2000). The reported variability of SLNB technique in the neoadjuvant setting raises questions as to its feasibility and accuracy. Other questions to consider are related to the effect of chemotherapy on lymphatic channels and whether chemotherapy similarly affects both sentinel and non-sentinel nodes. This study used single SLNB method with the methylene blue only. The researchers wished to evaluate the methylene blue absorption of SLNB procedure to early-stage breast-cancer patients after NAC.

## Materials and Methods

*Case selection*

Included in this study were patients with primary operable breast carcinomas diagnosed from 2016 to 2019 at Kasih Ibu Hospital, Surakarta and MNC Hospital, Jakarta affiliated to Sebelas Maret University Faculty of Medicine, Indonesia through a retrospective review of the surgical pathology report databases and medical record review.

The enrollment criteria of the post-NAC group and no-NAC group (control group) were as follows: 1) female; 2) confirmed diagnosed of breast carcinoma by Core Needle Biopsy and open biopsy; 3) with solitary lesion; and 4) no previous chemotherapy, endocrine therapy, radiotherapy or target therapy for the control group. The population is all stage I and II breast-cancer patients with clinically negative axillary lymph nodes. All of them, both post-NAC and control-group patients, had undergone SLNB with a single method using methylene blue conducted by surgical oncology consultants.

The data on the medical history and patient characteristics (including age, biopsy, tumor size, surgery, breast cancer type, injection location of methylene blue, and methylene blue absorption) were collected. The tumor size was presented as the maximum diameter of the main tumor mass under ultrasound.

*Statistical method*

The absorptions rate was defined as the proportion of pa¬tients with the detection of SLNs (blue results macroscopically on SLN using methylene blue only) among the total num-ber of patients who underwent SLNB in no-NAC and post-NAC groups.

The data were analyzed using IBM SPSS Statistics for Windows, Version 23.0 (Armonk, NY: IBM Corp.). The method used was the historical cohort study. The qualitative data were expressed as frequencies and percentages. Chi-square or Fisher’s exact tests were used to examine the relationship between qualitative variables as appropriate. The P-value <0.05 was considered statistically significant.

Consider the study where the goal is to assess the relative risk (RR) between methylene blue absorptions (no-NAC vs. post-NAC). After collecting the data from the total of 564 early-stage patients who were referred to surgical oncologists, the following was reported: samples were collected from 117 patients of stage-I and -II breast cancer with clinically negative axillary lymph nodes. The population was then grouped into two: those who had no-NAC (52 patients) and post-NAC (65 patients). SLNB procedures on each group were conducted by the periareolar or peritumoral injection of 2cc of methylene blue. The RR was calculated to determine the risk, or likelihood, of having methylene blue absorption. The calculated RR is reported using a 2x2 Table. Methylene blue absorption was assessed by using blue results macroscopically on SLN, and we calculated the percentages of the relative risk probability of the non-NAC group (control group) compared to the probability of the post-NAC group. The researchers also show the descriptive data about demographics, clinical data, surgery, and histopathology. 

*Ethical approval*

This study was approved by the Ethics Committee of Sebelas Maret University Faculty of Medicine, Indonesia. Ethical wise, patients’ consents are usually not required for data collection and research purposes if any steps involved in collecting patients’ information or research do not compromise with the quality of treatment and confidentiality of the patient. Throughout this research, patients’ confidentiality was maintained at the strictest level.

## Results

*Characteristics of patients of both groups*

The effects of neoadjuvant chemotherapy to the results of SLNB procedures can be assessed through blue color absorption of methylene blue on SLN. From the total of 564 early-stage patients who were referred to surgical oncologists, 117 patients were found to meet the criteria of inclusion, consisting of the post-NAC group (65 patients) and the control group (52 patients). The characteristics of the patients are shown in table 1 below. 

In this study, the highest number of patients in both groups was found to be included at the age range of 40 years or more, with 59 patients on the post-NAC group (91%) and 38 patients on the control group (73%). In this research, the histopathology type with the highest number of patients in both groups is Invasive Ductal Carcinoma (IDC), with 59 patients in the post-NAC group (91%) and 44 patients in the control group (85%). Most of the patients in both groups have T2-sized tumor, with 55 patients in the post-NAC group (85%) and 48 patients in the control group (92%). 

In the post-NAC group, most of the patients (54 patients/ 83%) underwent BCS to deal with their primary tumor, while in the control group; mastectomy for primary tumor was mostly performed to 41 patients (79%). Both groups also had similarity in the administration of SLNB procedures, with periareolar injection of methylene blue as the most common form of administration to 61 patients (94%) in the post-NAC group and 45 patients (87%) in the control group.

*Comparison of methylene blue absorptions between control and NAC groups*

We observed contradictory results for changes in the methylene blue absorption, with an increase observed in the control group and a decrease in the NAC group. Based on the bivariate analysis of the comparison of methylene blue absorption results on SLNB procedures, it was found that of 65 patients who had undergone NAC treatment (post-NAC) and SLNB procedures, 40 patients (61.5%) showed blue SLN results, while 38.5% were not blue SLN. Of 52 control-group breast-cancer patients, it was found that 47 patients (90.4%) showed methylene blue absorption on SLN. By using corrected Chi-square test (Fisher’s exact test), it was found that the p-value is 0.000 (P<0.05). It means that there is a significant relationship between post-NAC and control groups that have undergone SLNB procedures and absorption of methylene blue on SLN. The RR value equal to 0.522 was obtained. RR < 1 means that the risk of the methylene blue absorption in SLN is decreased by the NAC. 

*Factors that are likely to be associated with the decrease in the methylene blue absorption*

Based on the bivariate analysis, the tumor size (T1 and T2), operation (Mastectomy and BCS), injection location (Periaerola and Peritumoral Injection), and biopsy (core and open biopsy) are not correlated. It was found that 65 patients had undergone NAC treatment (post-NAC) and SLNB procedures. Of 52 control-group patients, it was found that 47 patients (90.4%) showed methylene blue absorption on SLN. By using the corrected Chi-square test (Fisher’s exact test), it was found that the p-value is 0.728 (tumor size vs methylene blue absorption), 0.552 (injection location vs methylene blue absorption), 0.065 (operation vs methylene blue absorption), and 0.38 (biopsy vs methylene blue absorption), which is greater than the alpha value (P>0.05). It means that there is no significant relationship between these factors.

## Discussion

Chemotherapy is claimed to cause lymphatic drainage damage because of the tumor cell’s apoptosis process. This event might cause decreased marker (radioactive solution and/or blue dye) absorption on sentinel lymph nodes (SLN). The reported variability of SLNB technique in the neoadjuvant setting raises questions as to its feasibility and accuracy. In this study, the researchers used methylene blue only and wished to evaluate the methylene blue absorption of the SLNB procedure on early-stage breast-cancer patients after neoadjuvant chemotherapy (NAC). This research found that the highest number of breast-cancer patients were on the age of 40 years or more (97 patients/ 82.9%). The histopathology type results of 103 patients (88%) indicated invasive ductal carcinoma (IDC), which is the same as the result of the research by Yarso et al., (2012) in Indonesia showing that from the 687 breast-cancer patients of 23-82 years old with the average age of 48.5 years, 89% of them have IDC. Most of the patients (106/ 90.5%) had undergone periareolar injection while peritumoral injection was given to 11 patients (9.5%). Borgstein et al., (2000) and Shimazu et al., (2002) compared peritumoral with periareolar blue coloring injection and concluded that periareolar injection is the ideal technique in identifying axillary SLN in the early-stage breast cancer. 

Various dyes studied for SLNB include isosulfan blue, patent blue, methylene blue, or fluorescein (Somashekhar et al., 2008). First, Simmons et al., (2003) described one for the first reports of successfully using methylene blue for SLN mapping. In this research, we have good results using the single method with methylene blue only. From 65 patients who had undergone NAC treatments and SLNB procedures, it was found that 40 patients (61.5%) showed positive blue SLN results. Of 52 control-group breast-cancer patients, it was found that 47 patients (90.4%) showed methylene blue absorption on SLN. P-value of 0.000 (P<0.05, significant) was obtained by using the Chi-square test with the RR of 0.522, meaning that Post-NAC patients had a tendency of decreased absorption of methylene blue. In the study of Mamounas et al., (2005), The largest multicenter report to date originates from the NSABP B-27 test in which NAC was administered to 428 patients with the identification rate found to be 84.8%. The metaanalysis study by Xing et al., (2006) shows that SLNB conducted after NAC to clinically node-negative breast cancer patients has an acceptable, but decreasing accuracy. In a single-institution study with the largest sample with SLNB after NAC by MD Anderson Cancer Center (MDACC) that compared SLNB’s accuracy after NAC (n = 575) with SLNB (n = 3.171), the SLN identification rate was found to be 97.4% on the NAC group and 98.7% on the SLNB-only group (p = 0.017) (Hunt et al., 2009). A prospective multicenter study from France (GANEA) reported that of 195 patients with T3N0 breast cancer undergoing lymphatic mapping with blue coloring and radiocolloid, the identification rate was found to be 90%.15 Clinically lymphonodi-negative patients before NAC have higher identification rate than clinically lymphonodi-positive patients (N1) (94.6% vs 81.5%; p = 0.008). In a prospective research (SENTINA test) Ozmen et al., (2006) of four prospective groups of a multicenter group study designed to evaluate the specific algorithm of SLNB procedures after NAC, the patients with clinical involvement of axillary lymph nodes before NAC converted into clinically-negative after undergoing SLNB and axillary dissection after NAC, the SLNB detection rate was 80.1%. 

In this study, we compare the factors that can influence methylene blue absorption, the tumor size (T1 and T2), operation (Mastectomy and BCS), injection location (Periaerola and Peritumoral Injection), and biopsy (core and open biopsy) that have no correlation with the SLNB detection rate (P>0.05). Many studies show these factors have no correlation with the SLNB detection rate. In the study of Ozmen et al., (2006), a tumor size larger than 2 cm (comparison of the T1 and T3 tumors) was associated with SLN positivity. In the study of Gokhan et al., (2012), tumor size and open biopsy are not associated with SLNB detection rate (P>0.05). A total of 2,206 patients were enrolled in the study of Wong et al., (2002), There were no statistically significant differences in the SLN identification rates or false-negative rates between patients undergoing excisional versus needle biopsy. In the study of Krammer et al., (2016) the preoperative biopsy method does not significantly impact SLN mapping with periareolar nuclide injection (P=0,4). The study of Boughey et al., (2015), shows that patient factors (age, BMI), tumor factors (clinical T or N stage), pathologic nodal response to chemotherapy, site of tracer injection and length of chemotherapy treatment do not significantly affect the SLN identification rate. 

To the best of our knowledge, this is the most comprehensive study to analyze the methylene blue absorption on SLN from SLNB procedures. We also suggest this method in neoadjuvant and SLNB breast-cancer studies to minimize the interference of SLNB and other confounding factors. Our study is limited in the analysis performed retrospectively at two institutions. There is a need for prospective studies with representative patient populations to confirm our results. 

In conclusion, neoadjuvant chemotherapy can cause the decrease of methylene blue absorption on SLNB procedures.

**Figure 1:**
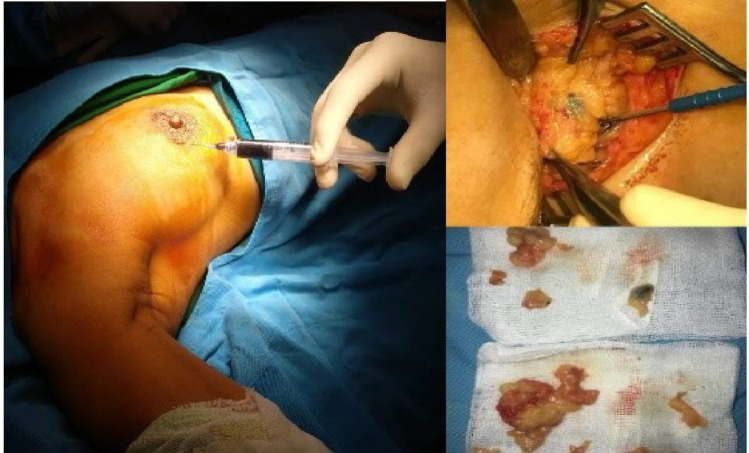
SLNB Procedure from Yarso KY, MD. Ph.D

**Table 1:** Characteristics of Control-Group and NAC-Group Patients

	Age		Biopsy		PA Result		T Status		Surgical procedure		Injection location	
	<40 yrs	>40 yrs	Core Biopsy	Open Biopsy	IDC	ILC	T1	T2	Mastec-tomy	BCS	Peri-tumoral	Peri-areola
Control Group (n: 52)V	14 (27)	38 (73)	52 (100)	0 (0)	44 (85)	8 (15)	4 (8)	48 (92)	41 (79)	11 (21)	7 (13)	45 (87)
Post-NAC (n: 65)	6 (9)	59 (91)	10 (15.4)	55 (84.6)	59 (91)	6 (9)	10 (15)	55 (85)	11 (17)	54 (83)	4 (6)	61 (94)

Data is presented as n (%)

**Table 2:** Comparison of Methylene Blue Absorption Results on SLNB Procedures

	Post-NAC (n:65) n (%)	No-NAC (n: 52) Control group n (%)	Relative Risk (RR)	*P*-value
Blue SLN	40 (61.5)	47 (90.4)	0.552	0
Not blue SLN	25 (38.5)	5 (9.6)		

**Figure 2:**
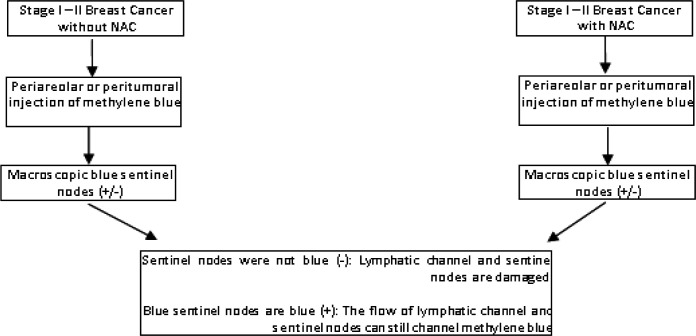
Research Flow

**Figure 3:**
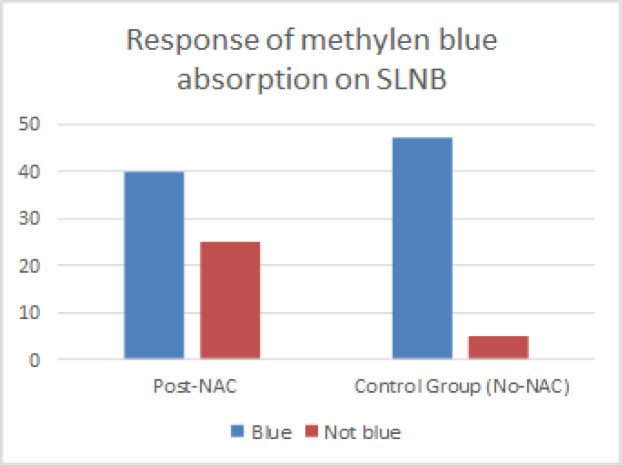
Distribution of Methylene Blue Absorption Response on Control and NAC Groups

**Table 3:** Mastectomy and BCS, Periaerola and Peritumoral Injection, Core and Open Biopsy have no Correlation

	Blue Nodes n (%)	Not Blue Nodes n (%)	*P*-value
Tumor size			
T1	10 (8.5)	4 (3.4)	
T2	78 (66.7)	25 (21.4)	0.728
Injection location			
Peritumoral Injection	9 (7.7)	2 (1.7)	
Periaerola Injection	78 (66.7)	28 (23.9)	0.552
Operation			
Mastectomy	43 (36.8)	9 (7.7)	0.065
BCS	44 (37.6)	21 (17.9)	
Biopsy			
Core Biopsy	51 (43.6)	11 (9.4)	
Open Biopsy	36 (30.8)	19 (16.2)	0.38
